# Prevalence of Microorganisms in Atherosclerotic Plaques of Coronary Arteries: A Systematic Review and Meta-Analysis

**DOI:** 10.1155/2022/8678967

**Published:** 2022-12-01

**Authors:** Iman Razeghian-Jahromi, Zahra Elyaspour, Mohammad Javad Zibaeenezhad, Soheil Hassanipour

**Affiliations:** ^1^Cardiovascular Research Center, Shiraz University of Medical Sciences, Shiraz, Iran; ^2^Cardiovascular Diseases Research Center, Department of Cardiology, Heshmat Hospital, School of Medicine, Guilan University of Medical Sciences, Rasht, Iran

## Abstract

**Background:**

In this systematic review and meta-analysis, the existence of pathogens in atherosclerotic plaques of coronary arteries was investigated in coronary arteries diseases (CAD) patients.

**Methods:**

This study was designed and implemented up to 31 August 2020. The findings present according to the PRISMA (Preferred Reporting Items for Systematic Reviews and Meta-Analysis) checklist. Two independent reviewers (I.RJ and S.H) performed a comprehensive search on four different English databases including PubMed, ISI, Scopus, and Embase. In order to assess the quality of the articles, a checklist prepared by The Joanna Briggs Institute (JBI) was used.

**Results:**

Finally, 44 studies were selected. The prevalence of different microorganisms in coronary arteries were as follows: *Aggregatibacter actinomycetemcomitans* (46.2%), *Campylobacter rectus* (43.0%), *Chlamydia pneumonia* (42.8%), *Cytomegalovirus* (29.1%), *Helicobacter pylori* (18.9%), *Herpes simplex* virus type 1 (5.9%), *Porphyromonas gingivalis* (42.6%), *Prevotella intermedia* (47.6%), *Tannerella forsythia* (43.7%), and *Treponema denticola* (32.9%).

**Conclusion:**

Based on the result of this meta-analysis, *Prevotella intermedia* and *Aggregatibacter actinomycetemcomitans* are the most common microorganisms in atherosclerotic plaques of coronary arteries and may have an important role in the development of atherosclerosis.

## 1. Introduction

Cardiovascular disease (CVD) is increasingly challenging people's health, irrespective of age, gender, and race. Coronary artery disease (CAD) is the main culprit in the growing burden of CVD [1]. CAD clinically appeared as the focal thickening of the intima layer due to the formation of atheroma [1]. Atheroma is the main content of atherosclerotic plaque constituting macrophages, cholesterol, smooth muscle cells, and dystrophic calcification [2]. Association of atherosclerosis with both innate and adaptive immunity is evidenced [3]. Chronic inflammation, endothelial dysfunction, and lipid accumulation in the vasculature are hallmarks of atherosclerosis [4].

Formation and development of atherosclerosis are feasible in the absence of traditional risk factors [4]. Surprisingly, nearly half of the patients with CVD are free from known cardiovascular risk factors such as hypercholesterolemia, hypertension, smoking, and obesity [5]. In order to reduce related morbidity and mortality, precise identification of all underlying reasons is of paramount importance.

Infections involve, at least in part, in CAD initiation and development [1]. Atherosclerosis may be originated from bacterial infection in terms of microbial symbiosis and inflammatory stimulus [6, 7]. Microbial agents contribute to the atherosclerosis process directly by infecting the vascular cells or indirectly by activation of inflammatory cytokines [4]. In this systematic review and meta-analysis, the existence of pathogens in atherosclerotic plaques of coronary arteries was investigated in CAD patients.

## 2. Materials and Methods

### 2.1. Study Design

The present study is a systematic review and meta-analysis investigating the existence of microorganisms isolated from atherosclerotic plaques of coronary arteries. This study was designed and implemented up to 31 August 2020. The findings present according to the PRISMA (Preferred Reporting Items for Systematic Reviews and Meta-Analysis) checklist.

### 2.2. Information Sources and Search Strategy

Two independent reviewers (IRJ and SH) performed a comprehensive search on four different English databases including PubMed, ISI, Scopus, and Embase. Specific search strategies were designed and used for each database. The included keywords were previous isolated bacterial species (*Aggregatibacter actinomycetecomitans, Chlamydiae pneumonia, Campylobacter rectus, Enteroacter hormaechei, Eikenella corrodens, Fusobacterium nucleatum, Fusobacterium necrophorum, Helicobacter pylori, Mycoplasma pneumonia, Porphyromonas endodontalis, Porphyromonas gingivalis, Prevotella intermedia, Prevotella nigrescens, Pseudomonas aeruginosa, Pseudomonas luteola, Streptococcus gordonii, Streptococcus mitis, Streptococcus mutans, Streptococcus oralis, Streptococcus sanguinis, Treponema denticola, Tannerella forsythia,* and *Veillonella*) along with relevant cardiovascular terms (Cardio^*∗*^, Cardiovascular, Cardiac, Heart, HF, Heart failure, Atherosclerosis, Athero^*∗*^, Arteri^*∗*^, Atherosclerotic, Coronary, atherosclerotic plaques, Endocarditis, Cardit^*∗*^, Aortic, Aort^*∗*^, Myocardial, and Myocardi^*∗*^).

### 2.3. Eligibility Criteria

Articles were imported into the EndNote software (version X7). Duplicate ones were automatically deleted from the dataset. Eligibility criteria were original studies on human samples published in English reporting microbial footprints in atherosclerotic plaques of coronary arteries. Studies published in other languages other than English, papers without full text, review articles, and articles presented in conferences as abstracts were excluded.

### 2.4. Quality Assessment

In order to assess the quality of the articles, a checklist prepared by The Joanna Briggs Institute (JBI) was used. The purpose of this appraisal is to assess the methodological quality of a study and to determine the extent to which a study has addressed the possibility of bias in its design, conduct, and analysis. All papers were evaluated on the basis of data relevance and methodological rigor.

### 2.5. Screening of the Articles

Primary searching of titles and abstracts was done by IRJ and ZE. They separately extracted data as well as evaluation of the articles' quality control. In case of ambiguity in one article, the principal investigator issued the final decision.

### 2.6. Data Extraction Form

A predefined checklist was prepared in order to extract data from all eligible articles. This checklist contained the author name, publishing year, country of origin, method of microorganism detection, and prevalence of each isolated microorganism.

### 2.7. Statistical Analysis

Heterogeneity between studies and their combination were checked by the Cochran test and *I*^2^ parameter, respectively. In the case of heterogeneity, a random effect model with the inverse variance method was used. Otherwise, the fixed effect model was used. All the analyses were performed by CMA statistical software (ver. 2).

## 3. Results

### 3.1. Description of Search

**Figure 1:**
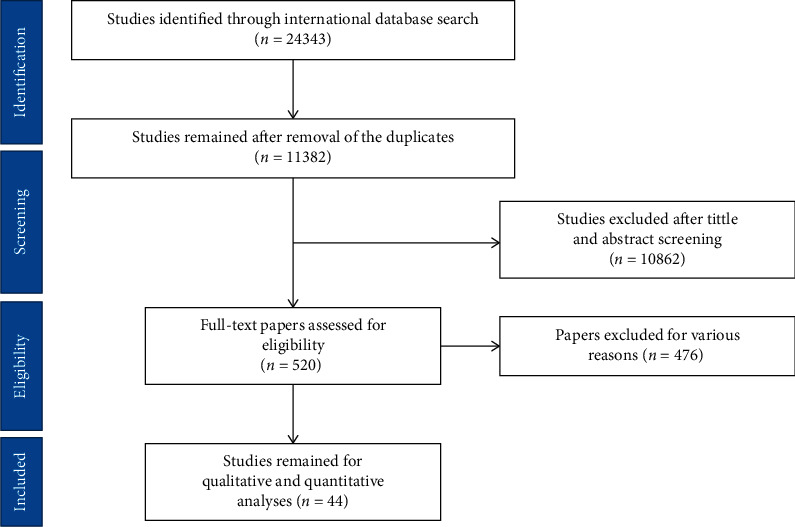
Flowchart of the included eligible studies in the systematic review.

Following searching of four defined databases (PubMed, ISI, SCOPUS, and Embase), 24343 articles were found which was decreased to 11382 after duplicate deletion. After screening their title and abstracts, 10862 articles were omitted owing to irrelevance to the study aim. The whole text of 520 papers was read and 44 were used for final analysis. It should be noted that references of the enrolled articles were screened as well to include eligible articles. The flowchart of the enrolled articles was depicted in Figure 1. The characteristics of included studies were demonstrated in Table 1.

### 3.2. Characteristics of the Included Studies

Based on geographical distribution, seven studies were conducted in Iran, five studies in Poland, five studies in India, three studies in Japan, four studies in Germany, four studies in Brazil, two studies in Korea, two studies in Turkey, two studies in the UK, and one study in Finland, Netherlands, Spain, Serbia, Belgium, France, Canada, Japan and USA, USA, and Denmark.

### 3.3. The Results of Meta-Analysis

**Figure 2:**
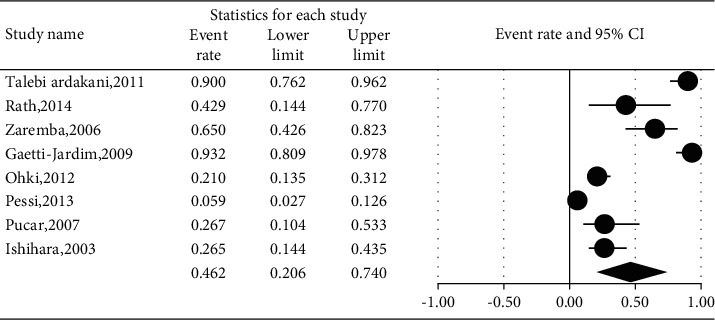
The prevalence of *Aggregatibacter actinomycetemcomitans* in atherosclerotic plaques of coronary arteries.

**Figure 3:**
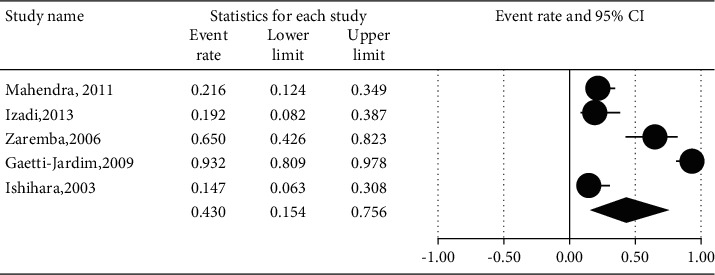
The prevalence of *Campylobacter rectus* in atherosclerotic plaques of coronary arteries.

**Figure 4:**
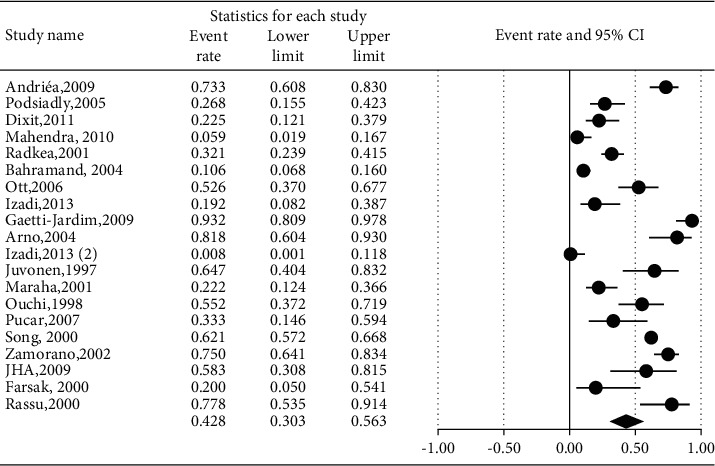
The prevalence of *Chlamydia pneumoniae* in atherosclerotic plaques of coronary arteries.

**Figure 5:**
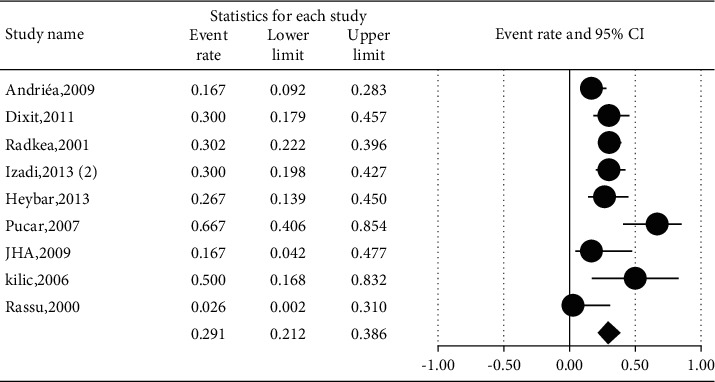
The prevalence of *Cytomegalovirus* in atherosclerotic plaques of coronary arteries.

**Figure 6:**
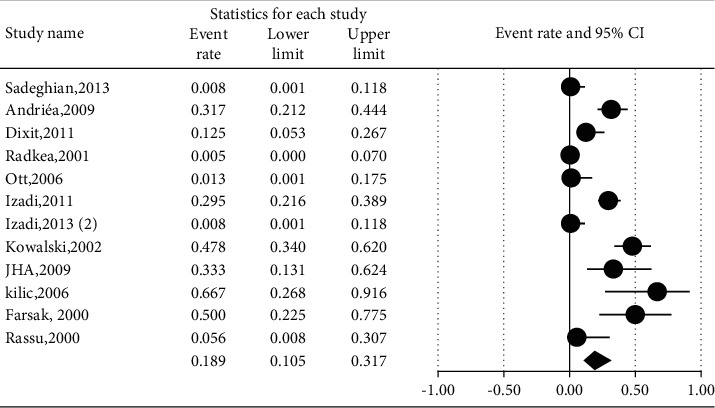
The prevalence of *Helicobacter pylori* in atherosclerotic plaques of coronary arteries.

**Figure 7:**
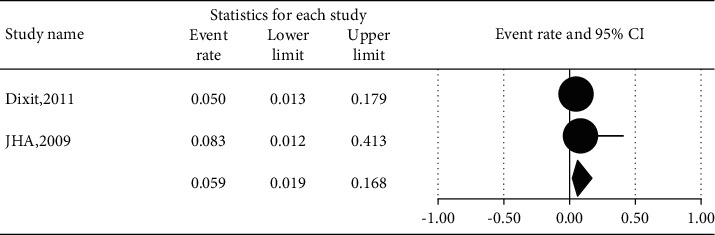
The prevalence of Herpes simplex virus type 1 in atherosclerotic plaques of coronary arteries.

**Figure 8:**
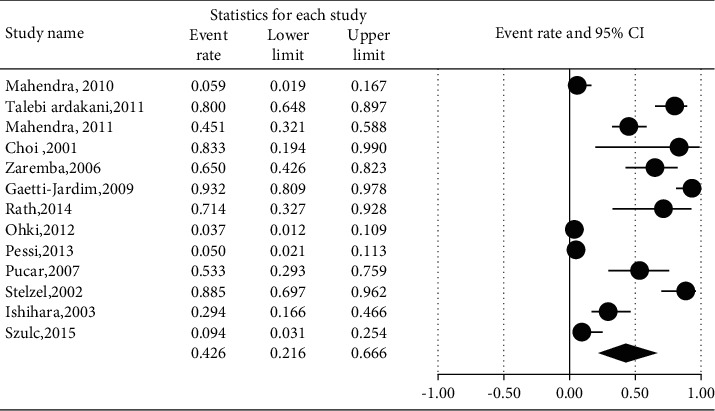
The prevalence of *Porphyromonas gingivalis* in atherosclerotic plaques of coronary arteries.

**Figure 9:**
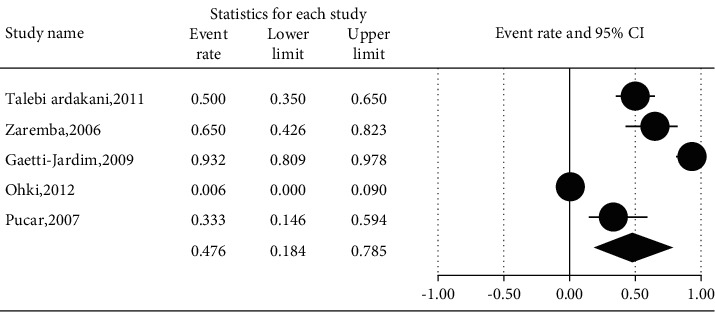
The prevalence of *Prevotella intermedia* in atherosclerotic plaques of coronary arteries.

**Figure 10:**
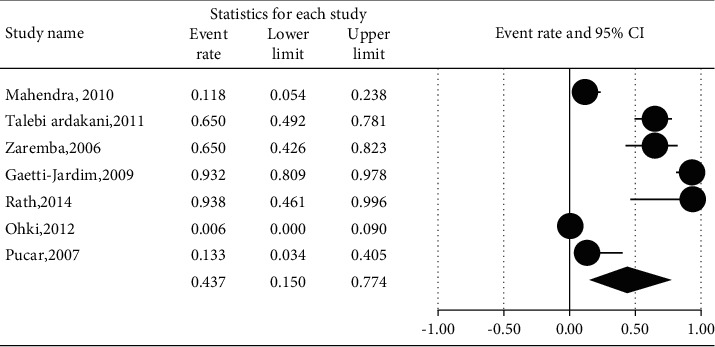
The prevalence of *Tannerella forsythia* in atherosclerotic plaques of coronary arteries.

**Figure 11:**
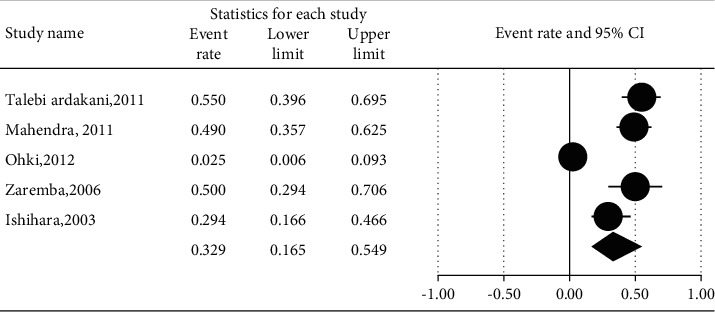
The prevalence of *Treponema denticola* in atherosclerotic plaques of coronary arteries.

The prevalence of different microorganisms in coronary arteries were as follows: *Aggregatibacter actinomycetemcomitans* (46.2%, 95% CI: 20.6–74.0, *I*^2^ = 92.2%, *P* value <0.001) (Figure 2), *Campylobacter rectus* (43.0%, 95% CI: 15.4–75.6, *I*^2^ = 91.7%, *P* value <0.001) (Figure 3), *Chlamydia pneumonia* (42.8%, 95% CI: 30.3–56.3, *I*^2^ = 92.4%, *P* value <0.001) (Figure 4), *Cytomegalovirus* (29.1%, 95% CI: 21.2–38.6, *I*^2^ = 56.7%, *P* value = 0.018) (Figure 5), *Helicobacter pylori* (18.9%, 95% CI: 10.5–31.7, *I*^2^ = 79%, *P* value <0.001) (Figure 6), *Herpes simplex virus* type 1 (5.9%, 95% CI: 1.9–16.8, *I*^2^ = 0.0%, *P* value = 0.667) (Figure 7), *Porphyromonas gingivalis* (42.6%, 95% CI: 21.6–66.6, *I*^2^ = 92.1%, *P* value <0.001) (Figure 8), *Prevotella intermedia* (47.6%, 95% CI: 18.4–78.5, *I*^2^ = 88.2%, *P* value <0.001) (Figure 9), *Tannerella forsythia* (43.7%, 95% CI: 15.0–77.4, *I*^2^ = 91.2%, *P*-value <0.001) (Figure 10), and *Treponema denticola* (32.9%, 95% CI: 16.5–54.9, *I*^2^ = 85.9%, *P* value <0.001) (Figure 11).

## 4. Discussion

At present, CAD is a worldwide cause of hospitalization and mortality [6]. Beside well-established risk factors such as genetic susceptibility, hypertension, hypercholesterolemia, and smoking, the risk of atherosclerotic vascular disease is increased by microbial infection [8]. For the first time, the association of microbial infection with atherosclerosis was demonstrated by Marek's disease virus. This virus induced atherosclerosis in chickens [9]. To substantiate, vaccination against this virus significantly protected chickens from atherosclerosis [10]. The human microbiome which mainly accumulates in the gut and oral cavity has an outstanding role to human health. Pathogens were found in thrombotic samples of MI patients [11]. Other than CVD, this huge microbial population is also involved in cardiovascular risk factors such as obesity and diabetes [12–15].

The potential of pathogens in promoting chronic disorders even in remote organs like colon cancers and atherosclerosis has been evidenced [16]. That is why chronic periodontal infection is in close relation to acute myocardial infarction (MI) and CAD [17, 18]. It may be interesting if you know that more than 275 oral bacterial species could enter the bloodstream through injured capillaries [19, 20]. Oral biofilm is in close proximity to the periodontal vasculature, and this makes the entrance and spread of bacteria feasible even to remote organs like the heart [21]. Indeed, in the case of inflammation (periodontitis or gingivitis), bacterial entrance is further smoothed due to increased permeability of the adjacent vessels [22]. Following bacterial access to the bloodstream, they could be localized in different parts of the human body, especially in sites with pathologic changes [23, 24]. In one study, subgingival plaques and different blood vessels of patients with atherosclerosis and periodontitis were sought in order to determine the frequency of periodontal bacteria. Findings revealed that the most and the least prevalent bacteria were *Tannerella forsythensis* and *Treponema* denticola, respectively. Also, the prevalence of *A. actinomycetemcomitans* and *Prevotella intermedia* were significantly different between subjects below and over 60 years of age [25].

The role of certain microbial infections such as *C. pneumoniae*, *P. gingivalis*, *A. actinomycetemcomitans*, *H. pylori*, influenza virus, and cytomegalovirus has been confirmed in the development of atherosclerosis in animal models [4]. Infection of vascular cells, detection of the microbes within the atherosclerotic plaque, and the development of atherosclerotic lesions after microbial infection in animal models reinforce the direct association of infection with atherosclerosis. On the other hand, indirect effects represent the emergence of cytokines and acute phase proteins followed by infection at nonvascular sites [4].

Infection with the Influenza virus increases the risk of the acute coronary syndrome and fatal MI [26]. Bacterial footprints were found from specimens of the heart valve and even aortic aneurysm [27]. A high amount of *S. mutans* has been detected concurrently in the heart valve samples, dental plaque, and saliva samples [28]. The presence of oral pathogens like streptococci and *Porphyromonas gingivalis* has been reported in atherosclerotic plaques of the carotid artery [29]. Also, bacterial genomes or their ribosomal DNA have been detected in atherosclerotic lesions of different arteries like aorta or coronary [30–34]. Nucleic acid or antigens of a wide range of pathogens, either bacteria or viruses, were found in atherosclerotic plaques [35–53]. Our studies show the presence of different pathogens (bacteria and virus) in the atherosclerotic plaques of coronary arteries. Unless herpes simplex virus type I, the existence of other pathogens is statistically significant. This may show the implication of a microbial center for the initiation and development of atherosclerotic plaque. However, this should be assessed by further comprehensive studies at the cellular and molecular level.

Several studies reported the presence of more than one pathogen in atherosclerotic plaques [29, 30, 37, 38, 40, 41, 46, 54, 55]. The simultaneous existence of some bacteria synergistically enhances their virulence [16]. Virulence factors of bacteria including fimbriae, degradative enzymes, exopolysaccharide capsules, toxins, and atypical lipopolysaccharides trigger the process of inflammation and affect vital organs like the cardiovascular system [56–58]. These adverse effects will be more detrimental in mixed infections [59–64].

The immune system is a major part of atherosclerosis progression. Microbial infection instigates immune responses [65]. Production of antibody is emerged after bacterial infections. This immune reaction increases the expression of inflammatory mediators, which in turn involves in the development of coronary thrombosis [65]. Also, microbial infections, either bacterial or viral, activate vessel-associated leucocytes [65]. The binding of bacterial-associated molecular patterns to Toll-like receptors triggers a signaling pathway, which upregulates some genes with a key role in atherogenesis (adhesion molecules in endothelial cells and inflammatory mediators in immune cells) [66]. Some bacteria like *P. gingivalis* accelerate atherosclerosis through enhancing the Th17 responses [3]. A specific immune response and aortic inflammation are raised with chronic oral *P. gingivalis* infection [67].

Activation of platelets by bacteria results in thrombosis formation [1, 68]. Some bacteria like streptococci are endothelial-adhesive. This facilitates embedding to the damaged heart valves and subsequent thrombosis induction [65]. Consequent inflammation and vascular changes may terminate to the functional impairment of both endothelial cells and smooth muscles of coronary arteries [69, 70].

It seems that the entry of bacteria and other microbiome populations into the bloodstream is a continuous flow which inevitably leads to a surge in the expression of inflammatory cytokines and chemokines. These factors could be drivers of CAD [71]. For example, it was shown that bacterial lipopolysaccharide (LPS) upregulates LDL levels, increasing the risk of CAD [72]. Indeed, the remnants of bacteria like DNA or membrane phospholipids provoke CAD via modulating adipose or vascular tissues [6]. However, it was reported that the development of atherosclerotic lesions is largely accelerated by live organisms rather than heat-killed ones or their LPS [73]. Reports on successful culturing of pathogens after their isolation from atheroma only existed about *C. pneumoniae* and *E. hormaechei* [74–77].

Overall, it does not clearly understand if initiation or progression of atherosclerosis is dependent on the presence of microbes. In an interesting study, the bacterial profile of atherosclerotic plaques was compared between symptomatic and asymptomatic CAD patients. Neither the amount of bacterial DNA nor microbial composition was not different between the two groups. It was concluded that the vulnerability of plaques may be influenced by other factors [1]. *In vitro* studies demonstrated that invasive strains of *P.gingivalis* help foam cell formation which is a pivotal step in the evolution of atherosclerotic plaques [78]. Although the role of bacteria like *Chlamydia pneumoniae* has been suggested in this process [79], antibacterial therapy did not address atherosclerotic-related complications [80].

In spite of acceptable evidence on the role of microbial infection in atherosclerosis, which is derived mostly from animal models, it seems that assigning a definitive determining role as equal as other cardiovascular risk factors for the development of atherosclerosis in humans is debated and there are numerous questions that need to be answered in this area [4].

Our study tried to cover almost all the published data on this subject. However, we should consider the possibility of unintentional mistakes to include qualified papers among hundreds of screened papers.

## 5. Conclusion

Undoubtedly, microbes contribute substantially to human hemostasis. However, access to these elements in some sites may stimulate adverse events. It is important to elucidate the role of microbes in CAD as atherosclerosis imposes a huge health burden. This needs to be addressed in order to save resources for the rainy days and unprecedented diseases.

## Figures and Tables

**Table 1:** Basic characteristics of included studies.

Order	Author/year	Country	Method of detection	Prevalence of isolated microorganism
1	Juvonen et al. /1996	Finland	PCR, IHC	*Ch. pneumoniae* (65%) Total sample = 18
2	Ouchi et al. /1997	Japan	PCR, IHC	*Ch. pneumoniae* (44%) by IHC *Ch. pneumoniae* (55%) by PCR Total lesion = 29
3	Song et al. /1999	Korea	IHC	*Ch. pneumoniae* (57%) Total sample = 7
4	Radke et al. /2000	UK	PCR	CMV (30%), *Ch. pneumoniae* (32%), *H. pylori* (0%), Cytomegalovirus + *Ch. pneumoniae* (5%) Total lesion = 106
5	Farsak et al. /2000	Turkey	PCR	*Ch. pneumoniae* (10%), *H. pylori* (50%), *Ch. pneumoniae*, and *H. pylori* (20%) Total lesion = 10
6	Rassu et al. /2000	Italy	PCR, IHC	CMV (18%), *H. pylori* (40%) Total lesion = 18
7	Choi et al. /2001	Korea	PCR	*P. gingivalis* (100%) Total lesion = 2
8	Kowalski et al. /2001	Germany	PCR	*H. pylori* (42%) Total = 42
9	Maraha et al. /2001	Netherlands	PCR, IHC	*Ch. pneumoniae* (22% by PCR, 60% by IHC), *M. pneumoniae* (1%) by PCR Total sample = 95
10	Stelzel et al. /2002	Germany	PCR	*P. gingivalis* (88.5%) Total sample = 26
11	Zamorano et al. /2002	Spain	PCR	*Ch. pneumoniae* (76%) Total lesion = 76
12	Ishihara et al. /2003	Japan, USA	PCR	*P. gingivalis* (29%), *P. intermedia* (9%), *E. faecalis* (9%), *P.* nigrescens (9%), *A. actinomycetemcomitans* (3%), *C. rectus* (3%), *T. forsythia* (3%), *P. endodontalis* (1.9%), *T. denticola* (1.9%), *F. nucleatum* (0%) Total lesion = 51
13	Bahrmand et al. /2004	Iran	PCR	*Ch. pneumoniae* (81%) Total lesion = 22
14	Arno et al. /2004	UK	PCR	*Chlamydia* species (71%) Total lesion = 31
15	Podsiadły et al. /2005	Poland	PCR	*Ch. pneumoniae* (26%) Total lesion = 41
16	Ott et al. /2004	Germany	PCR, clones culture	*Chlamydia* species (51.5%) by PCR, *Ch. pneumoniae* (%0), *Ch. trachomatis* (0%), *H. pylori* (0%), mycoplasma species (0%) by PCR *Ch. pneumoniae* (71%) by clones culture Total lesion = 38
17	Zaremba et al. /2006	Poland	PCR	*A. actinomycetemcomitans*, (5%) *P. intermedia* (15%), *P. gingivalis* (50%), *E. corrodens* (15%), *C. rectus* (20%), *T. forsythia* (25%), *T. denticola* (30%), *F. nucleatum* (25%) Total lesion = 20
18	Zaremba et al. /2006	Poland	PCR	*Periodontitis bacteria* (55%) Total lesions = 20
19	Kilic et al. /2006	Turkey	PCR	*H. pylori* (23%), CMV (23%) Total sample = 30
20	Pucar et al. /2007	Serbia	PCR	*P. gingivalis* (53.33%), *A. actinomycetemcomitans* (26.67%), *P. intermedia* (33.33%), *T. forsythia* (13.33%), Cytomegaloviruses (66.67%), *Ch. pneumoniae* (33.33%) Total lesion = 15
21	Reszkaa et al. /2007	Poland	PCR	*Ch. pneumoniae* (27.5%), *M. pneumoniae* (15%), *H. pylori* (80%), herpes simplex viruses (67.5%) Total lesion = 40
22	Dabiri et al. /2008	Iran	PCR, DIF (direct immunofluorescence)	*Ch. pneumoniae* (19%) Total lesion = 26
23	Andriéa et al. /2009	Germany	Immunhistochemistry	*Ch. pneumoniae* 73%, HP in 31%, CMV in 16%, and EBV in 40%. Total lesions = 60
24	Gaetti-Jardim et al. /2009	Brazil	PCR	*A. actinomycetemcomitans*, *F. nucleatum*, *P. gingivalis*, *P. intermedia*, *P. nigrescens*, and *T. forsythia*, in 94.9% of the lesions. Te pathogens frequently found in coronary sites. Total lesion = 44
25	Mahendra et al. /2009	India	PCR	*T. denticola* (49.01%), *E. corrodens* (27.45%), *C. rectus* (21.51%), *P. gingivalis* (45.10%) Total patients = 51
26	Hem et al.	India	PCR	*C. pneumoniae* (58%), *H. pylori* (33%) Total lesion = 12
27	Silvia et al. /2010	Brazil	PCR	*P. gingivalis* (50%), *P. intermedia* (16%), *E. faecalis* (16%), *P. nigrescens* (16%), *A. actinomycetemcomitans* (6%), *C. rectus* (6%), *T. forsythia* (6%), *P. endodontalis* (3%), *T. denticola* (3%), *F. nucleatum* (0%) Total lesion = 30
28	Mahendra et al. /2010	India	PCR	*T. forsythia* (11.8%), *P. gingivalis* (5.9%), *P. gingivalis* fimA (5.9%), *P. nigrescens* (5.9%) Total lesion = 51
29	Mahendra et al. /2010	India	PCR	*T. denticola* (49.01%), *C. rectus* (21.51%), *P. gingivalis* (45.10%) Total lesion = 51
30	Jegier et al. /2010	Japan	PCR	Fungal (40%), albicans (58%), mycotics (48%) Total lesion = 40
31	Koren et al. /2010	USA	PCR	Chryseomonas (100%), firmicutes (63.8%), bacteroidetes (11.7%), proteobacteria (15.4%), actinobacteria (6.4%) Total lesion = 14
32	Damodar et al. /2011	Belgium	PCR	CMV (30%), herpes simplex virus type 1 (5%) and herpes simplex virus type 2 (42.5%), *H. pylori* (12.5%), *Ch. pneumoniae* (22.5%) Total lesion = 40
33	Talebi ardakani et al. /2010	Iran	PCR	*A. actinomycetemcomitans* (90%), *P. gingivalis* (80%), *P. intermedia* (50%), *T. forsythia* (65%), *T. denticola* (55%), *F. nucleatum* (30%) Total lesion = 20
34	Izadi et al. /2011	Iran	PCR	*H. pylori* (29.5%) Total lesion = 105
35	Ohki et al. /2012	Japan	PCR	*A. actinomycetemcomitans* (21.0%), *P. gingivalis* (3.7%), *P. intermedia* (0.0%), *T. forsythia* (0.0%), *T. denticola* (2.5%) Total lesion = 81
36	Sadeghian et al./2013	Iran	PCR	*H. pylori* (0%) Total lesion = 30
37	Calandrini et al. /2013	Brazil	PCR	Betaproteo bacteria (21.7%), pseudomonadaceae (26.1%), alphaproteobacteria (13.0%) Total lesion = 35
38	Izadi et al. /2013	Iran	PCR	*Ch. pneumoniae* and *H. pylori* (0%), CMV (30% in acute MI and 3.33% in nonacute MI) Total lesion = 16
39	Heybar et al. /2013	Iran	PCR	CMV (14.9%) Total sample = 110
40	Pessi et al. /2013	France	PCR	*Streptococcus* sp. mainly *S. mitis* group (%72.3), *A. actinomycetemcomitans* (5.9%), *P. gingivalis* (5.0%), endodontic bacteria (78.2%), periodontal bacteria (34.6%) Total lesion = 9
41	Saroj et al. /2014	India	PCR	*A. actinomycetemcomitans* (42.86%), *P. gingivalis* (71.43%), tannerella species (100%) Total lesion = 7
42	Filho et al. /2014	Canada, Brazil	DNA amplification and cloning	Actinobacteria (10.0%), bacteroidetes (17.5%), firmicutes (17.5%), fusobacteria (5.0%), proteobacteria (45.0%), *Prevotella intermedia* 2.5%, synergistetes (0.0%), gracilibacteria (GNO2) 2.5% Total lesion = 40
43	Szulc et al. /2015	Poland	PCR	*P. gingivalis* (9.4%) Total lesion = 32
44	Hansen et al. /2016	Denmark	DNA FISH	Positive bacteria (81%) identified a total of 55 different bacterial species. Total lesion = 22

## Data Availability

The data used to support the study are available from the corresponding author upon request.
